# Does temporal irregularity drive prediction failure in schizophrenia? temporal modelling of ERPs

**DOI:** 10.1038/s41537-022-00239-7

**Published:** 2022-03-17

**Authors:** Maria Karanikolaou, Jakub Limanowski, Georg Northoff

**Affiliations:** 1 National Technical University of Athens, Applied Mathematics and Physical Sciences, Athens, Greece; 2 Faculty of Psychology and Center for Tactile Internet with Human-in-the-Loop, Technische Universität Dresden, Dresden, Germany; 3 Institute of Mental Health Research, University of Ottawa, Ottawa, ON Canada

**Keywords:** Schizophrenia, Schizophrenia

## Abstract

Schizophrenia subjects often suffer from a failure to properly predict incoming inputs; most notably, some patients exhibit impaired prediction of the sensory consequences of their own actions. The mechanisms underlying this deficit remain unclear, though. One possible mechanism could consist in aberrant predictive processing, as schizophrenic patients show relatively less attenuated neuronal activity to self-produced tones, than healthy controls. Here, we tested the hypothesis that this aberrant predictive mechanism would manifest itself in the *temporal irregularity* of neuronal signals. For that purpose, we here introduce an event-related potential (ERP) study model analysis that consists of an EEG real-time model equation, eeg(t) and a frequency Laplace transformed Transfer Function (TF) equation, eeg(s). Combining circuit analysis with control and cable theory, we estimate the temporal model representations of auditory ERPs to reveal neural mechanisms that make predictions about self-generated sensations. We use data from 49 schizophrenic patients (SZ) and 32 healthy control (HC) subjects in an auditory ‘prediction’ paradigm; i.e., who either pressed a button to deliver a sound tone (epoch a), or just heard the tone without button press (epoch b). Our results show significantly higher degrees of temporal irregularity or imprecision between different trials of the ERP from the Cz electrode (N100, P200) in SZ compared to HC (Levene’s test, *p* < 0.0001) as indexed by altered latency, lower similarity/correlation of single trial time courses (using dynamic time warping), and longer settling times to reach steady state in the intertrial interval. Using machine learning, SZ vs HC could be highly accurately classified (92%) based on the temporal parameters of their ERPs’ TF models, using as features the poles of the TF rational functions. Together, our findings show temporal irregularity or imprecision between single trials to be abnormally increased in SZ. This may indicate a general impairment of SZ, related to precisely predicting the sensory consequences of one’s actions.

## Introduction

The brain is governed by continuously changing dynamics that has a strong impact on perception, decision-making, cognition and behavior^1,2^. How such dynamics shape and are manifested in neural activity remains largely unknown, though; therefore, we also do not know whether and how they can be linked to abnormal neuronal processes as observed, for instance, in schizophrenia. One issue is that methodologically, we tend to conceive of dynamics as mere noise that needs to be eliminated from the data. For instance, differences in the timing of different single event-related trials are often neglected and considered pure noise that is to be eliminated from the data^3^. This neglects that the very same temporal differences between single trials may contain important information^4,5^ including temporal precision. Importantly, temporal precision has been shown to be impaired on the behavioral level of schizophrenia with its neural correlates remaining unclear, though^6,7^. Addressing temporal precision on the neural activity level and how it affects the prediction of auditory event-related potentials in healthy and schizophrenia subjects is the main goal of our study.

Prediction is a key of the brain’s neural activity^8–10^. Neural response metrics of sensory attenuation may provide us with the most flexible model basis to connect our dynamic mechanisms with Laplace transformation and Transfer Function equations to extract feature information^11^. In a nutshell, there is a neural signal related to the prediction of an incoming external stimulus, this is called the empirical prior or predicted input. The latter is subsequently compared with the actual input with the degree of their difference resulting in the prediction error. Subjects suffering from schizophrenia are well known to suffer from a prediction failure^12,13^ as they remain unable to yield a proper empirical prior or predicted input^14–16^. We here address the question whether such prediction failure is related to temporal imprecision between different single trials: if subsequent single trials are temporally heterogeneous and hence imprecise, it may be difficult to yield the predicted input or empirical prior at the “right” point in time relative to the time point of the incoming actual input. Accordingly, we are interested in probing whether schizophrenia subjects distort the “normal prediction process” through temporal imprecision in their neural activity.

How can we detect the temporal basis of prediction failure in schizophrenia?^17–22^ Investigating the effects of predicted input or empirical prior in EEG is ideally suited to investigate its temporal precision (or lack thereof); this is the main goal of our study. For that purpose, we develop a novel Transfer Function model (TF) that allows us to map the temporal precision or imprecision of single trials in event-related potentials (ERP) of EEG (See Methods for details). Combined with a typical auditory ‘prediction’ paradigm (Ford et al., 2014; see Methods), this allows us to investigate temporal imprecision on a neural level and how it impacts and shapes prediction:

In this task, Ford et al. (2014)^23^ had shown that SZ have reduced N1 suppression and smaller LRPs preceding button presses to deliver tones, which the authors interpreted as evidence for abnormal predictive mechanisms. Here, we hypothesize that schizophrenia subjects exhibit temporal imprecision in their single trial event-related potentials (ERP) during specifically those moments in the single trial where prediction is required. More generally, we suppose that prediction failure in schizophrenia is, at least in part, related to temporal irregularity or imprecision between individual trials.

## Results

### Timing of ERP—temporal irregularity of single trials in SZ

**Fig. 1:**
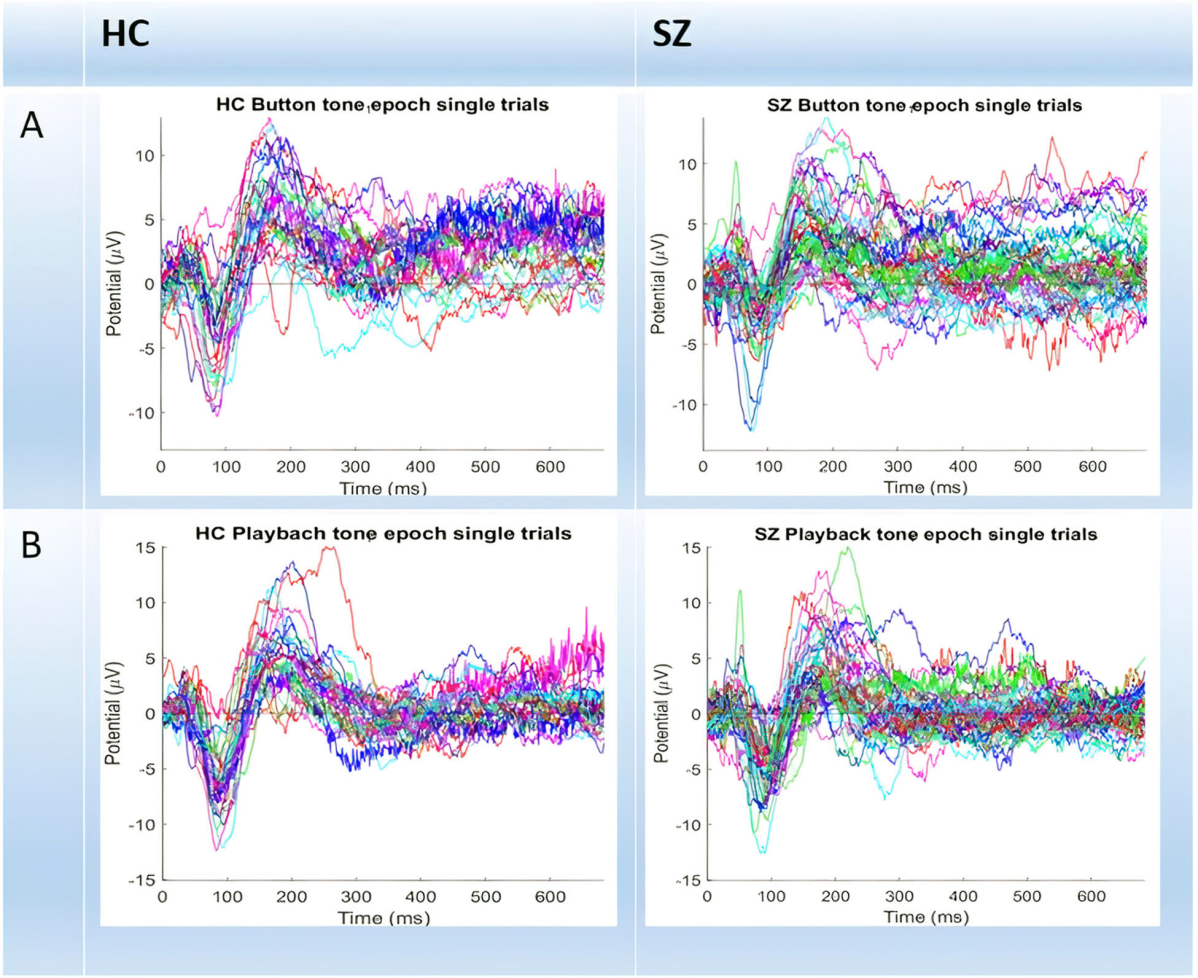
Schizophrenic Patients and Healthy Controls, during Button-press tone (A) and Playback passive tone (B). The descriptives in the temporal domain are supported by our findings which detect the differences in the temporal domain.

In a first step, we plotted all single trials in both groups (see Fig. 1). It can be seen that especially in the feedback component (P200), SZ subjects show higher variability between single trials which, visually, seems to largely result from different temporal courses among the different trials—this strongly suggest higher temporal irregularity or imprecision from trial to trial in SZ. In our subsequent analyses, we aim to operationalize and quantify such temporal imprecision following the measures introduced in the method. Therefore, we focus on the timing rather than the amplitude of the typical ERP components like N100 and P200 as elicited in our paradigm^24,25^ (see Supplementary material for epoch-averaged results on amplitude).

The visual observations of temporal imprecision between single trials are confirmed in quantitative analysis (Table 1). Our findings about the timing or latency of Healthy Controls and Schizophrenic patients show a N100 latency [mean ± SD] of HC [90.1 ± 6.7 ms] vs SZ [90.2 ± 7.4 ms], a P200 time of HC [185.8 ± 21.2 ms] vs SZ [161.4 ± 17.8 ms], where in the P200 latency we obtained a significant difference between Healthy Controls and Schizophrenic patients (Levene’s test: *p* < 0.0001), while N100 timing was not significantly different. This is in accordance with previous findings showing similar dissociation of N100 and P200 in SZ which concerned the amplitude, though^26–30^. Hence, our observation extends this N100-P200 dissociation to their timing, with shorter P200 latency in Schizophrenia^31–35^.

**Table 1:** Time characteristics (ms) calculated by TF models [mean ± SD].

	Epoch	SZ patients	Controls
N1 latency	a	90.4 ± 7.7	91.0 ± 7.8
	b	90.1 ± 7.5	88.4 ± 3.7
P2 latency	a	165.4 ± 17.5	185.4 ± 20.0
	b	158.5 ± 18.1	187.5 ± 22.0

More specifically, comparing epochs a and b, i.e., Button-press Tone vs Playback Tone, we again obtained significant difference in the timing of P200: epoch a showed a P200 time of HC [185.4 ± 20.03 ms] vs SZ [165.4 ± 17.5 ms]. While for epoch b, we obtained a P200 time of HC [187.5 ± 22.03 ms] vs SZ [158.5 ± 18.15 ms]. In contrast to P200, N100 latency was not significantly different, either when comparing a and b epochs, or when comparing HC vs SZ groups. These differences of the timing or latency strongly suggest larger temporal imprecision between single trials in the P200 of schizophrenia.

### Probing temporal irregularity—temporal course of different trials

**Fig. 2:**
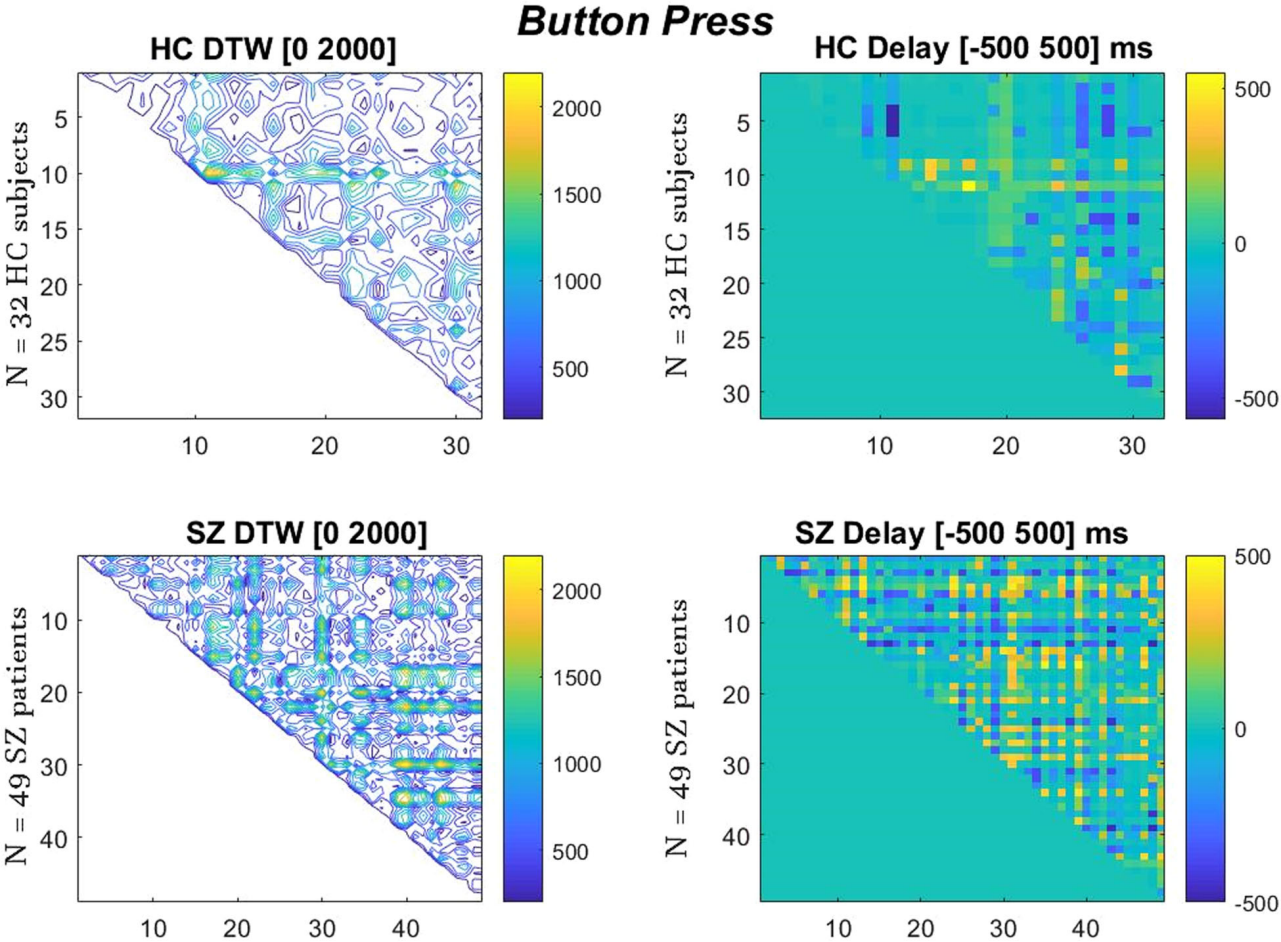
Dynamic Time Warping (DTW) (left) and Delay (right) results of ERP signals’ alignment. Inter-trial group comparison for HC group of 32, SZ group of 49 during a epoch (Button-press). The color bars show the DTW distance of each signal pair comparison and the color bars of Delay show the degree of timing correlation of each signal pair. We observe significantly higher DTW distance values in SZ (*p* = 0.005) and also higher Delay in SZ (*p* < 0.0001). DTW _**SZ**_ = 677.1 (CI = 652.0, 702.1, *p* = 0.005). DTW _**HC**_ = 600.7 (CI = 567.3, 634.1, *p* = 0.005). Delay _**HC**_ = 74 ms (CI = 65, 84, *p* < 0.0001). Delay _**SZ**_ = 119 ms (CI = 112, 126, *p* < 0.0001).

**Fig. 3:**
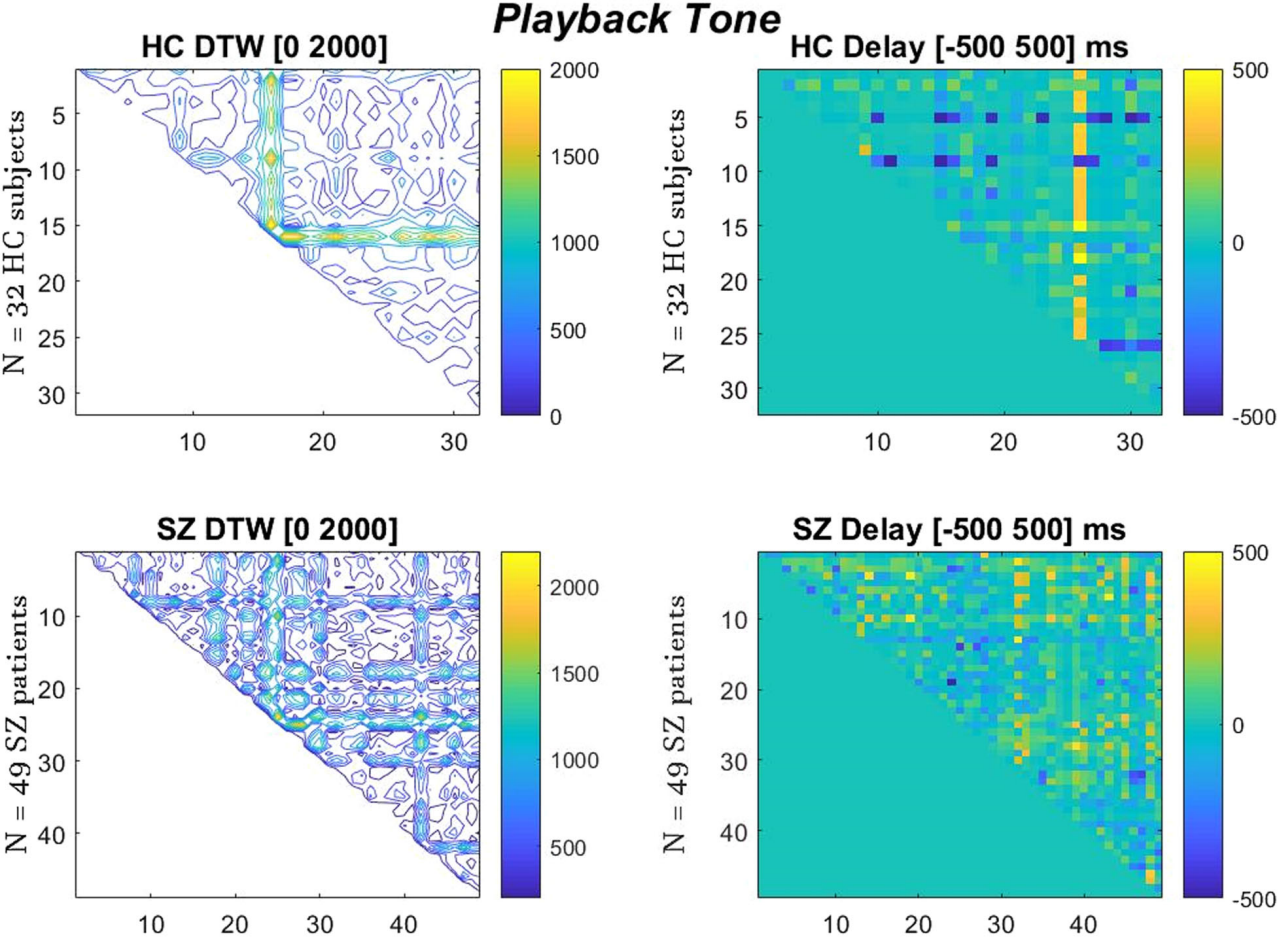
Dynamic Time Warping (DTW) (left) and Delay (right) results of ERP signals’ alignment. Inter-trial group comparison for HC group of 32, SZ group of 49, during b epoch (Playback Tone). We again observe significantly higher DTW distance values in SZ (*p* < 0.0001) and also higher Delay in SZ (*p* = 0.046). DTW _SZ_ = 528.2 (CI = 507.7, 548.8, *p* < 0.0001). DTW _HC_ = 444.6 (CI = 413.4, 475.8, *p* < 0.0001). Delay _HC_ = 67 ms (CI = 57.5, 76.9, *p* = 0.046). Delay _SZ_ = 78 ms (CI = 73.5, 82.7, *p* = 0.046).

HC showed significantly higher correlation (DTW) in the time courses among their single trials when compared to SZ. Figures 2 and 3 present the DTW and delay results show differences (along with the 95% Confidence Intervals and *p* values) between HC and SZ, with the latter presenting greater DTW distance, thus indexing lower degrees of temporal similarity between their ERP signals. Spatial metrics indeed capture the temporal discontinuity in the SZ graphs, as we can see from the most close-to-zero values on the right graph of Healthy Controls and the less dense contour on the left graph. Together, these results further confirm that the time courses of the single trials and their delays were more different among each other in SZ than in HC—SZ are more temporally irregular or imprecise in the time courses and the delays of their single trials than HC.

At first, we aimed to study the Inter-Trial Coherence (see Supplementary Material for ITC EEGLAB results), where we noticed a lower temporal similarity among SZ group successive trials in low gamma frequencies compared to HC subjects^36–39^. Thus, we used DTW metrics to further demonstrate the similarity between the signal sequences.

DTW performs latency contrasts by a stretching algorithm that gives us the time-domain distance distortion path needed to align the signals in terms of phase and speed. While DTW finds the matching relationship between every subject’s ERP with one another (group inter-trial greater similarity means minimum DTW distance), the finddelay function reveals again the best temporal alignment within the HC and SZ groups trials, where it simply measures the temporal correlation in the samples series signal of the group combinations. In the Figs. 2, 3 below we present the visualization of the DTW and delay matrices of the two groups.

**Fig. 4:**
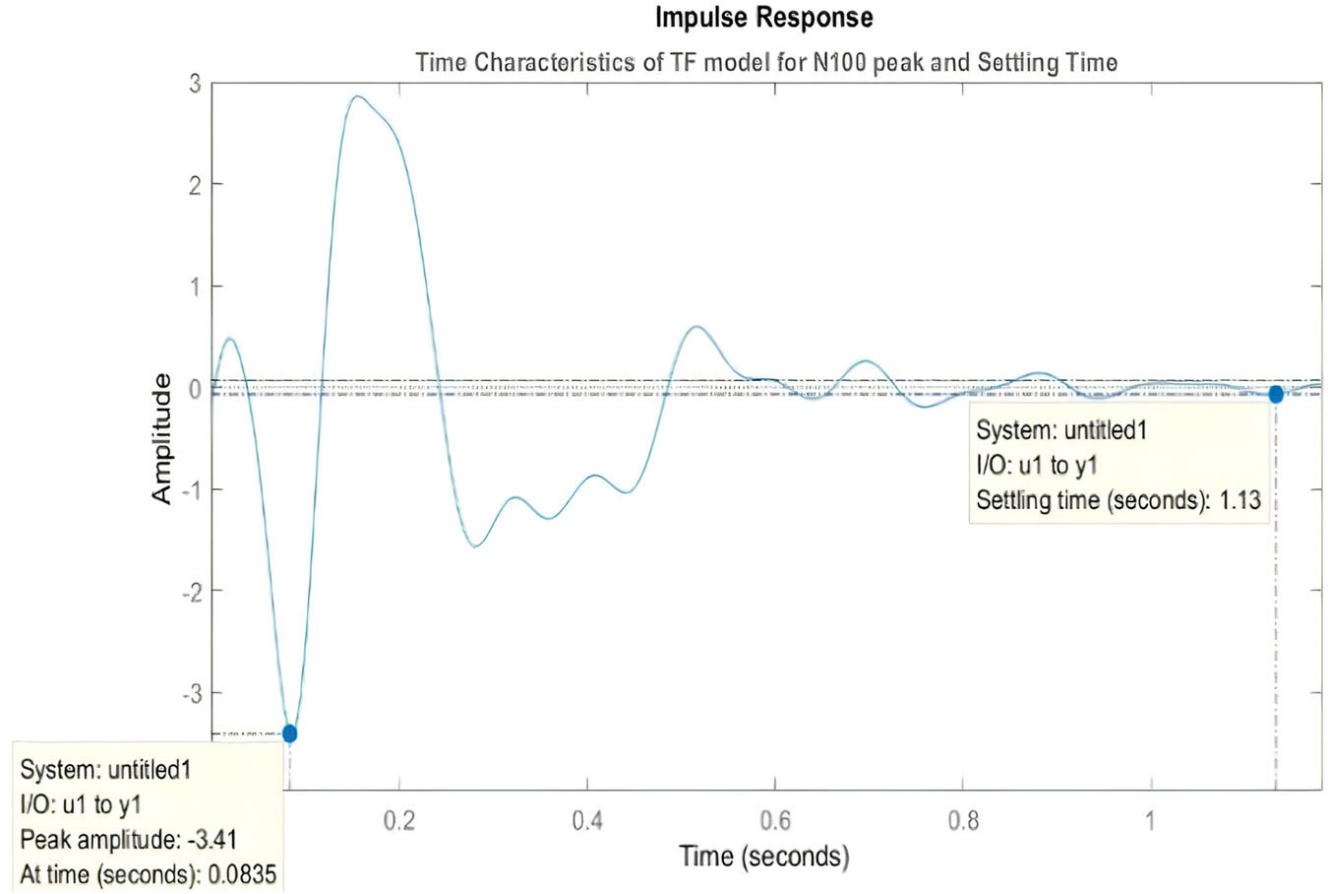
Transfer Function model simulation allows for temporal characteristics calculations (Settling Time and N100 peak time) of attenuation ERP components. The figure is produced by the “impulse” function of every TF model that has been calculated to fit each ERP.

Is temporal imprecision between trials related to their relationship, that is, the temporal carry-over of the previous trial to the subsequent one? In that case, one would expect that the time between the trials, i.e., the inter-trial interval is affected as it is key in mediating the temporal relationship from one trial to the one—this has also been designated as stimulus-rest interaction^40^. For that, we simply calculated in MATLAB the “lsiminfo” of our TF models to get what we describe as “settling time”, that is, the time neural activity needs to settle into a steady state in the intertrial interval following the exposure to the stimulus (see Methods for details and Fig. 4).

Settling times exhibited a significant difference among HC and SZ [mean ± SD], with *p* < 0.0001 through a Levene’s *t*-test. Specifically, HC showed shorter settling time [1.02 ± 0.18 s] than SZ [1.20 ± 0.28 s].

**Fig. 5:**
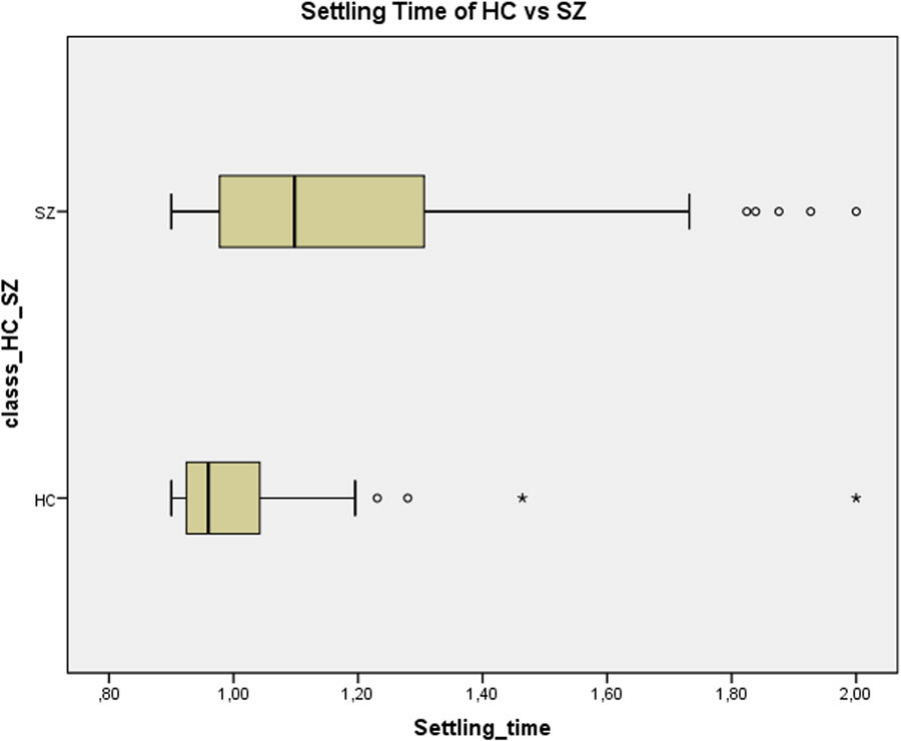
SPSS chart boxplot of Settling Time (ST) variance in each group. Schizophrenic patients reveal longer Settling time as well as higher SD [1.20 ± 0.28 s] than Healthy Controls [1.02 ± 0.18 s].

Hence, SZ reveal a longer “latency” in the intertrial interval’s return to a steady-state. That is further supported by the observation of greater ST variance in SZ than in HC (HC ST Variance of 0.03 while SZ ST Variance was 0.08) (Fig. 5).

The longer settling time in SZ and its higher variability across trials strongly suggest that neural activity in SZ takes longer and more variable time durations to return to a steady state after the exposure to the external stimulus. Given that the time interval between different stimuli (e.g., the intertrial interval) is key for the subject to predict the next incoming stimuli, longer and more variable settling times related to the previous stimulus may dent into and thus impair the prediction, including temporal precision, of the subsequent stimulus.

### Temporal irregularity—high classification accuracy

Does the temporal irregularity allow to classify SZ as distinct from HC? To construct the classifiers, after we have obtained the frequency model representations of each signal, of all HC and SZ subjects, we use the component vectors in the denominator, whose magnitude in the s-plane describe the distance of the point s from the pole—(see Methods for details).

Looking up to the Eqs. 3 and 4, we can analytically observe the correspondence between the Inverse Laplace solved time equation and the TF frequency equation. The poles of the latter contain the temporal information as seen in the exponential constant of Eq. 4 (real part of poles feature) and the frequency information as seen in the argument of the sin and cos functions of Eq. 4 (imaginary part of poles feature).

**Fig. 6:**
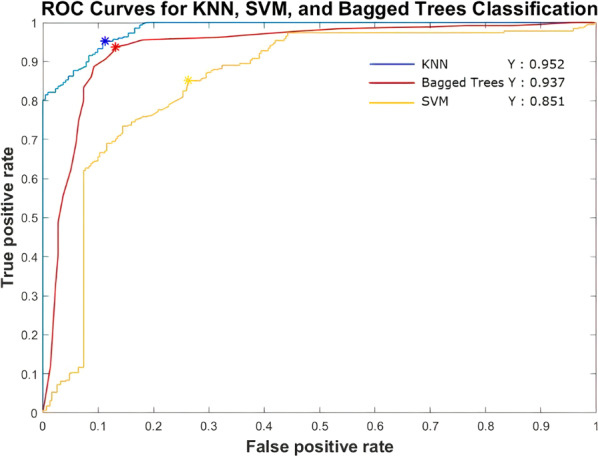
Receiver Operating Characteristic (ROC) curves of our Classifiers. They successfully classify the data of our TF models in the groups of HC or SZ.

To classify the two groups, SZ and HC subjects, we only chose those with whose frequencies are above 5 Hz, in order to avoid “noise” in our features. Classifiers, based on our feature selection, estimate for each target instance, 0 for HC, 1 for SZ, depending on the classification kernel, the greatest probability. Consequently, they decide about the point origin, among the two groups, successfully classifying ~92% of the data if all features are used (Table 2). The classification methods used are KNN (K-Nearest Neighbors), Bagged trees and SVM (Support Vector Machines), while their ROC (Receiver Operating Characteristic) curves demonstrate their results (Fig. 6). Together, the high classification accuracy means that temporal imprecision really signifies the ERP in SZ as distinguished from HC.

**Table 2:** Classifiers’ accuracy and confusion matrices.

Classification technique	Confusion matrix			Validation accuracy
Weighted KNN 2/3 features	0	387	47	0.919 92%
	1	39	587	
		0	1	
Bagged Trees 2/3 features	0	385	49	0.898 90%
	1	59	567	
		0	1	
Support Vector Machines 1/3 features	0	217	95	0.801 80%
	1	58	398	
		0	1

## Discussion

We took a novel, multi-methodological approach to analyzing ERP data related to abnormal predictive mechanisms in SZ^41–43^, by looking at their temporal features; i.e., their temporal regularity or precision from trial to trial. Our data show significantly higher temporal irregularity across trials in SZ, especially in the P200, a component that is closely related to prediction, i.e., empirical prior. The assumption of temporal imprecision is further supported by the correlation of the time course between the different trials, i.e., dynamic time warping, which showed higher distance between trials in SZ than HC.

In order to probe whether the temporal imprecision of single trials is related to dynamic features extending across the single trial itself, we calculated the settling time in the intertrial interval, that is, the time needed to get back to a steady-state. This yielded indeed significantly longer settling times and higher variability in SZ than HC; that, in turn, strongly impacts the empirical prior of prediction as required in our paradigm in the intertrial intervals. Finally, we show that temporal imprecision between single-trial ERP yield high classification accuracy with values over 90%. Together, our findings demonstrate that temporal irregularity or imprecision is a key factor in mediating prediction failure in SZ.

SZ showed higher temporal irregularity in particular the P200 which, given our paradigm, is closely related to prediction and its empirical priors. Such temporal imprecision between trials is further confirmed by our DTW analyses. This marks temporal imprecision in ERP of SZ which corresponds well to temporal imprecision as observed on the behavioral level^6^. Future analyses are needed to investigate whether such temporal imprecision of ERP is related to abnormal intrinsic neural timescales, like Trial-to-trial variability (TTV) investigating the prestimulus and poststimulus neural responses dynamics^44^.

A recent study observed prolonged intrinsic neural timescales in SZ during task states and, importantly, during the transition from rest to task^7^. If the intrinsic neural timescales are too long, they may render the timing or temporal course of ERP-related components imprecise as for enhanced temporal precision shorter timescales are needed. Hence, future studies may want to relate the intrinsic neural timescales to the time course of ERP, i.e., their temporal precision across trials.

We demonstrated that neuronal activity during the intertrial interval showed longer and more variable settling times in SZ after exposure to the stimulus^45^. This suggests a yet unclear dynamic deficit in SZ as they are no able to return to their initial steady state (or resting state) in proper time. Their neural activity does not seem to susceptible to change as it can also be observed on the opposite side during the transition from rest to task^7^. At the same time, the intertrial interval also provides the prestimulus period for the next time; this is relevant as predicted inputs (or “empirical priors”) are generated during the prestimulus period. Prolonged and more variable settling times in the intertrial interval may consecutively affect the timing of the empirical prior which, analogously may then also be more imprecise and variable.

Given that our paradigm included a strong predictive component, the empirical prior itself may be strongly affected by the partial overlap from the preceding stimulus. Over time, this may lead to an incorrect and temporally imprecise empirical prior with a high subsequent prediction error—the SZ subjects thus lose their adaptive capacities to flexibly predict and react to novel stimuli.

In sum, we have demonstrated that SZ show higher degrees of temporal irregularity across single trials of ERP. This is related to abnormalities in latency, dissimilarity between time courses, and longer and more variable settling times in the intertrial period. We conclude that temporal imprecision across single trials may be a key factor in driving the well-known prediction failure in schizophrenia.

## Methods

### Subjects

We process the auditory ERP signals of 32 Healthy Controls (HC) and 49 Schizophrenic patients (SZ), data which were retrieved from the Kaggle database, from the following dataset: “Basic Sensory Task in Schizophrenia” [https://www.kaggle.com/broach/button-tone-sz]. Some of the patients were diagnosed with DSM-IV schizophrenia (*N* = 23) and some with schizoaffective disorder (*N* = 3), based on the Structured Clinical Interview for DSM-IV (SCID). Additional data were analyzed from the 2nd dataset of the study [https://www.kaggle.com/broach/buttontonesz2].

University of California at San Francisco Institutional Review Board and San Francisco Veterans Affairs Medical Center approved the study, and all participants provided written informed consent.

### EEG task

The analysis was performed on two conditions acquired by Ford et al. 2014, reflecting a typical auditory prediction task. In the first condition (Button tone), 100 tones (80 dB sound pressure level, 1000 Hz) were self-generated through button-press by the subjects, every 1–2 s. This temporal sequence of tones was preserved and reproduced during the second condition (Playback tone); i.e., participants only listened to tones but did not press any buttons. In Ford et al.’s study, subjects also completed a third condition (button press without tone), which was irrelevant to the present question and therefore omitted.

### EEG data acquisition and processing

EEG data had been recorded from 64-scalp sites and 8 external sites using a BioSemi ActiveTwo system, digitized with a sampling frequency of 1024 Hz. EEG time series of 81 subjects (~9216 ms) were separated into the three conditions of 3000 ms epoch’s duration each one, time-locked to button presses (coincident with tone onset). The EEG epochs were corrected from artifacts for voltages exceeding ±100 μV at all scalp sites [Ford et al. 2013]. The experiment was conducted by Ford et al. with funding from the National Institute of Mental Health (NIMH grant number R01MH058262).

In this paper, we use the Cz channel data (in the future we will analyze the other channels as well), and we deconstruct the 3000 ms epochs to new intervals of 700 ms epoch’s duration for a and b condition, corresponding to Button press sound tone (epoch a) and Playback tone (epoch b), since in the 3rd condition (Button alone) no evoked potentials were present. We apply a lowpass Butterworth filter to smooth our ERPs in order to be able to apply properly the mathematical calculations (Transfer Function transformation, Dynamic Time Warping processing) described analytically in the following section. While we were interested in EEG inter-trial coherence (ITC) as well, along with our ERP simulations in MATLAB (ver. R2018a), in order to study the signals’ coherence, we used EEGLAB, the results of which were compared to pseudotrials’ ITC. SPSS has also been used for our statistical analysis (IBM Corp. Released 2015. IBM SPSS Statistics for Windows, Version 23.0. Armonk, NY: IBM Corp.).

### Transfer Function model (TF)

We develop the Transfer Function models (TF) by applying the Laplace transform to analyze this input-output relationship and we aim to detect differences between the waveforms (Fig. 4). The system (output is represented by ERP) receives a disturbance (input is modeled by a delta Dirac function, coincident with the trigger onset), changing the variables that describe it, and then returns to the natural steady state, like Rest-Stimulus modulated activity^46,47^. Based on mathematical control theory, receiving as input, X(s), a time series with only initial t_0_ onset value, and as output, Y(s), the ERP response of each subject, the computational mechanisms provide us with a TF model for every subject’s response of our EEG concatenated epoch arrays. With the appropriate MATLAB Laplace and TF functions we can efficiently calculate for every subject’s dynamical system a unique TF simulated response, each one of 12th degree, in order to have consistent fitting models (see Figs. 1–3 in Supplementary Material for details). The TF model is a ratio of two polynomials, in frequency domain, which as complex numbers, the Numerator and Denominator, have the following mathematical parameters: Real part, Re(z), Imaginary part, Im(z), and Magnitude, ||z||, of each complex number. These frequency characteristics of the complex s-plane are later translated to time domain characteristics, like settling time (ST), and also the time envelope constant in EEG(t) equations below.

In this model, we present the TF filter-form equation in our time series analysis, and we use the formula characteristics to further analyze how patients with schizophrenia and healthy controls process and filter repetitive stimuli and reveal any existent deficit. Without loss of generality, the TF provides a transformation basis of the unknown complicated differential equation of our neurophysiology system to a solvable algebraic equation. Through the damping ratio of the Inversed-Laplace solved time wave equations we can detect differences in the attenuation ERP components (Fig. 4).

The concept of the Transfer Function block diagrams represents the input-output relation for complex systems’ dynamics and describes high-order dimensional systems governed by differential equations, while it is a rational function of the complex variable, s. The Transfer Function is defined as the ratio of Output to Input, providing us itself with the notion of “gain”, being a measure of how the system amplifies or attenuates a signal that is impressed upon it. Thus every ERP dynamic’s Output system is simply the product of the Input plus the Transfer Function, where input is simulated as a Dirac function trigger on the onset of the stimulus time series. Taking into account the impulse input, we can simulate it as a delta function δ(t), therefore the Impulse Response will be calculated, from Y(s) = X(s)• H(s) (see Figs. 1, 2 in Supplementary Material for details).

When we have a multisensory input, the outcome of our action is “tuned”, in order to discriminate between self-Button press (active, self-initiated) and externally generated (passive) auditory triggers^19–21,24,48–57^.

We can see in Fig. 4 the use of our ERP TF model simulation that allows high temporal precision in analysis. We will present our results about how it calculates the N100 and P200 latencies for every model, and along with the Settling Time parameter (see below), the model provides all the temporal characteristics of every ERP waveform, enhanced or attenuated, after the trigger auditory impulse. Latency variability is provided by our TF model in a threefold manner, calculating at first N1/P2 latencies of every ERP model, then Settling Time of every ERP and then all the model parameters (time constants of envelope EEG(t) signal, see Eqs. 4, 5 in the following section) will be used in the classifier.

In electronic circuit analysis, a high-order control system undergoes a transient phase when it receives an input disturbance. Similarly, our “tuned” system has its own TF function, characterized by its settling time until it returns to its steady state, and presents a peak amplitude as well. The Transfer Function is defined as the ratio of Laplace transform of output response to Laplace transform of input (excitation). Equation (1) is a rational function with m poles and n zeros expressed in the complex variable s,

s = σ+ jω as follows:

1 $$H\left( s \right) = \frac{{Num(s)}}{{Den(s)}} = \frac{{b_ns^n + b_{n - 1}s^{n - 1} + \cdots + b_1s + b_0}}{{a_ms^m + a_{m - 1}s^{m - 1} + \cdots + a_1s + a_0}}$$

The transfer function provides a basis for detecting important system response characteristics without solving the complete complex differential equation, as demonstrated below in Eq. 2.

2 $$\begin{array}{ll}y^{(n)} + c_{n - 1}\;y^{(n - 1)} + \cdots + c_2\ddot y + c_1\dot y + c_0y = d_mu^{(m)} \\\qquad+\, d_{m - 1}u^{(m - 1)} + \cdots + d_2\ddot u + d_1\dot u + d_0u\end{array}$$

We need high precision, in order to obtain the most realistic simulation of our system by the transfer function. We confirm that the fit of our properly smoothed ERPs is in absolute accordance with the N1 and P2 evoked potentials. The increasing number of poles increases the complexity of our modeling transfer function, and ensures the suitability of the model dynamics. The algorithm is solved repeatedly, until the solution reaches the desired accuracy.

Once the expression of the Transfer Function for each subject is found in s-domain, we decompose the equation, in terms of Partial Fraction Expansion, where z_n_ are the zeros and p_m_ the poles of our transfer function, as shown in Eq. 3.

3 $$H\left( s \right) = \mathop {\sum }\limits_i^m \frac{{z_n}}{{s - p_m}}$$

### Settling Time and EEG(t) model

Every TF model Impulse Response supply us with the temporal characteristics of P200 and N100 time information and the Settling Time (ST) as we can see in Fig. 4. We also present the simulation of two random models, one for a SZ subject, one for a HC subject, with a Transfer Function of 12th degree, (~95% fitting percentage of model) in order for the comparison of the real ERP and the fitting model to be demonstrated (see Fig. 2 in Supplementary Material).

Temporospatial dynamics of rest and task states metrics are suggested for abnormalities in Schizophrenia in analogy with task-related activity and resting state^7^. The resting-state activity (RSA) of the system is related to ST as also rest-stimulus modulated activity of brain regions (auditory, cerebellum) is driven by auditory cues, given with passive listening or beeping tapping (tapping task with auditory sound)^40,58^. Transfer function models relate the rise time of the oscillation with the settling time of the actual “settling down” of the transient oscillation, while computationally they are calculated by a simple function where response is settled down when |y(t) - yfinal| becomes less than 2%. Settling time (ST) reflects the power by which the attenuation takes place, defined in circuit analysis as the time that the system needs to reach to its steady state again, after the damping caused by an external trigger. Greater settling time means that the system presents greater “resistance” in restoring energy. For an EEG neural network model that is conducting the input external signals and goes through a transient response we obtain each TF model which also gives us its Settling Time. In control systems theory, it is a measure of the quality of “control” of the system, as it clearly calculates the time it needs to integrate the “filtering” of the triggering energy it receives, and finally bounces or shifts back to its intrinsic or ‘natural frequencies’. The stability of the “control circuit” is well-reflected in the ST parameter that indexes the time the system needs to return to its natural steady-state oscillations.

Now, we can present an innovative temporal model as well, introducing a unique time-dependent expression for each ERP signal, for HC and SZ subjects correspondingly, which can be obtained by applying the Inverse Laplace transform and solving each H(s) equation in time domain. For our system, the zeros and poles, of which most are complex conjugates, effectively define the components of our system differential equation, while the denominator polynomial in terms of s of the transfer function is known as the characteristic polynomial.

We decompose our TF functions from the “z, complex plane” of Eq. 3 form, by applying the inverse Laplace transform and thus extract the time dependent signal expression for one HC subject, where we can also observe the cosine and sine arguments in Hz, in terms of the EEG β, α, δ rhythms (Eq. 4), which can be plotted in a time interval (Eq. 4, successfully represents Fig. 3 in Supplementary Material as an EEG(t) solution-expression of its unique TF ERP model):

4 $$\begin{array}{ll}EEG_{control}\left( t \right) = e^{ - 23.4t}\left[ {0.576 \cdot \cos \left( {30.6t} \right) + 1.53 \cdot \sin \left( {30.6t} \right)} \right]\\\qquad\qquad\qquad +\, e^{ - 7.6t}\left[ {0.45 \cdot \cos \left( {15.5t} \right) + 0.18 \cdot \sin \left( {15.5t} \right)} \right]\\\qquad\qquad\qquad +\, e^{ - 4.9t}\left[ { - 1.36 \cdot \cos \left( {9.2t} \right) + 2.4 \cdot \sin \left( {9.2t} \right)} \right]\\\qquad\qquad\qquad+\, 14.4 \cdot e^{ - 54.8t} + e^{ - 4.1t}\left[ {3.7 \cdot \cos \left( {5.8t} \right) + 3.2 \cdot \sin \left( {5.8t} \right)} \right]\\\qquad\qquad\qquad +\, e^{ - 2.5t}\left[ { - 5.1 \cdot \cos \left( {2.5t} \right) + 4.3 \cdot \sin \left( {2.5t} \right)} \right] - 12.7 \cdot e^{ - 13.2t}\end{array}$$

In order to see the solution’s steps of an H(s) equation part and its passage to the EEG(t) equation we hereby describe the Inverse Laplace application:

5 $$\begin{array}{l}\frac{{a + bj}}{{s - (c + dj)}} + \frac{{a - bj}}{{s - (c - dj)}}\\ \downarrow \begin{array}{*{20}{c}} {{{{\mathrm{Inverse}}}}} \\ {{{{\mathrm{Laplace}}}}} \end{array}\\ e^{ - ct}[ \pm 2a \cdot \cos (dt) \pm 2b \cdot \sin \left( {dt} \right)]\end{array}$$

### Dynamic Time Warping (DTW)

In order to measure temporal regularity/irregularity ore precision/imprecision of ERP’s in the empirical data, we compare their time course by measuring their distance. For that purpose, we use dynamic time warping (DTW). Roughly, DTW allows to correlate the time courses between single trials at each time point: similar and thus more regular and precise time courses of single trials should yield higher correlation and thus less temporal distance between trials. Combined with the delay metric that measures the temporal delays obtained in DTW, this provides insight into the degree of temporal regularity/irregularity of single trials of the ERP.

DTW is applied in the first 300ms of our signal, in order to minimize the noise of our results as well as the pinching effect. We perform DTW, which is using chess moves, to find the optimized minimum mapping distance of the time series points (warping path). Each two ERP signals compared will be distorted so that they are almost aligned on a common time axis, and that way DTW provides us with the similarities between the signals. The lowest cost optimal path gives the minimum DTW distance, and the best possible alignment.

Dynamic Time Warping (DTW) allows probing and validating the assumption of temporal irregularity in the ERP of schizophrenia. For that specific temporal purpose, we use DTW in combination with a samples delay metric (finddelay MATLAB function as to calculate how advanced is one signal from another, and then samples (lags) are transformed to ms). Inter-trial coherence is studied by us in EEGLAB before we performed DTW measures, and greater coherence was found indeed in healthy controls than in Schizophrenia patients (see Supplementary Material for details) while we also cross-checked our findings using pseudotrials, that is, trials without stimulus as derived from intertrial intervals to control for the impact of spontaneous activity fluctuations over the course of time^5,59^.

### Classification

Machine learning identification of EEG features is using signal processing approaches and mathematical modeling techniques to predict and make a decision in diagnostic classification^60,61^. For that purpose, we constructed classifiers to capture specifically the temporal features of ERPs. We probed whether all our Transfer Function measures together (TF model is characterized by its Settling Time, in the time domain, whereas in the frequency domain TF is defined by its complex numbers’ real part and imaginary part) provide high classification accuracy and also tested each of them by itself. This yielded the highest classification accuracy for all temporal measures being put together—that reached a value larger than 0.9 reflecting high accuracy (Table 2). Together, our classification results show the importance of the temporal components of the ERP in classifying and distinguishing HC and SZ, both in time domain (see Results for details in ST) and s-domain (classifiers’ accuracy). More generally, it demonstrates the high relevance of temporal irregularity/imprecision across trials in ERP of SZ as distinct from HC.

## Supplementary information

Supplementary Material

## Data Availability

All raw EEG data that were used in this study were available at: https://www.kaggle.com/broach/button-tone-sz. All other relevant processed data and scripts are available from the corresponding author on request.
