# Supplementary material for: Does temporal irregularity drive prediction failure in schizophrenia? temporal modelling of ERPs
Source: Schizophrenia (Heidelb). 2022 Mar 17;8(1):23. doi: 10.1038/s41537-022-00239-7 (PMC8931057; doi:10.1038/s41537-022-00239-7)
Supplement: Supplementary file 1 — Supplementary Material [file 41537_2022_239_MOESM1_ESM.docx]

# Supplementary Material


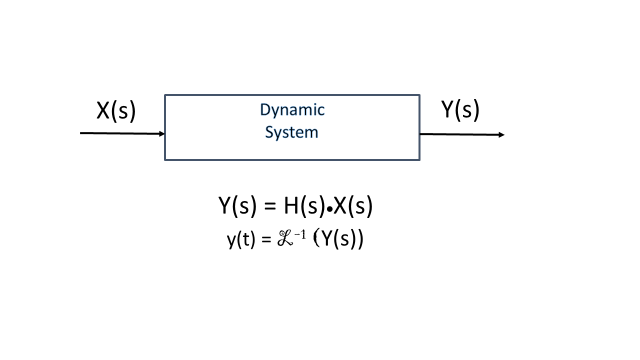


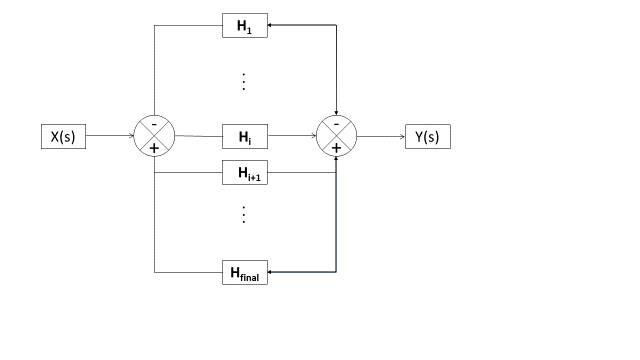


1. Procedure of frequency – time domain transition and mathematical solution of Laplace equation. Hypothetical block diagram implemented by the physiological system, receiving Input trigger, X(s), transferring it to Y(s) Output (ERP) through a unique H_final_ TF model calculation. Multiplication in the Laplace (frequency) domain X(s)•Y(s) is the operation of convolution in time domain of x(t)*y(t).


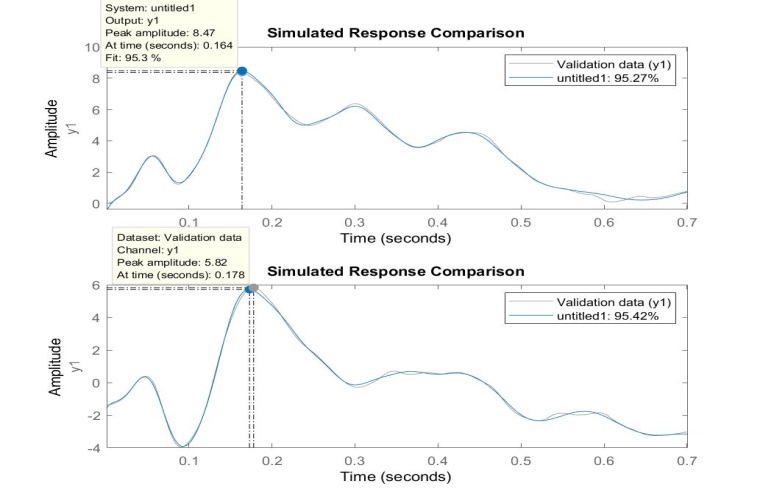


1. TF models (blue) simulated over real ERP signals (black). One SZ signal (up) and one HC signal (down) with fitting percentage over 95% for both models. While we cannot obviously detect differences, the TF models provide us with distinguishable equations for HC and SZ.


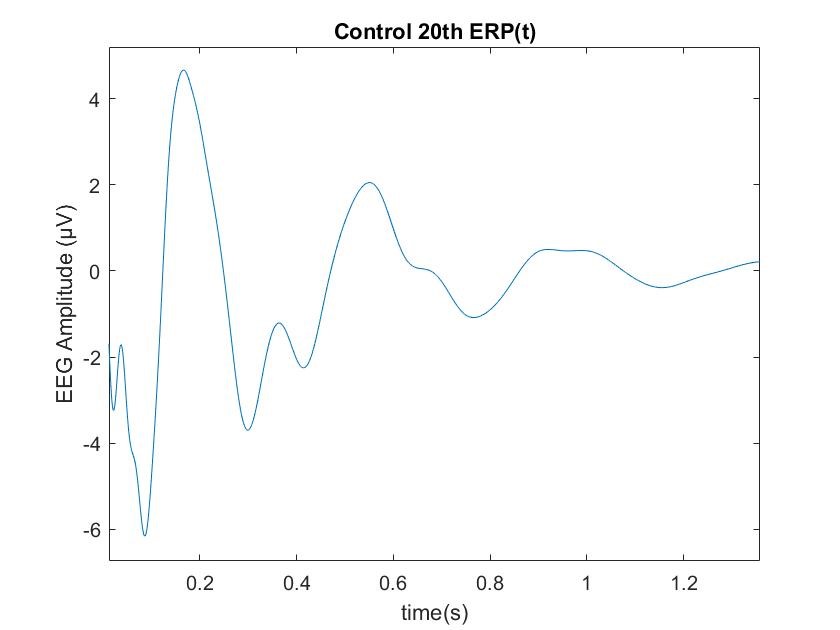


1. Equation 4 plotted in a time interval. The H(s) equation (Eq.3) for the corresponding ERP is solved (for some TF models) by applying the Inverse Laplace transformation, in order to exhibit the innovative temporal models that could constitute a time basis analysis for our future paper.


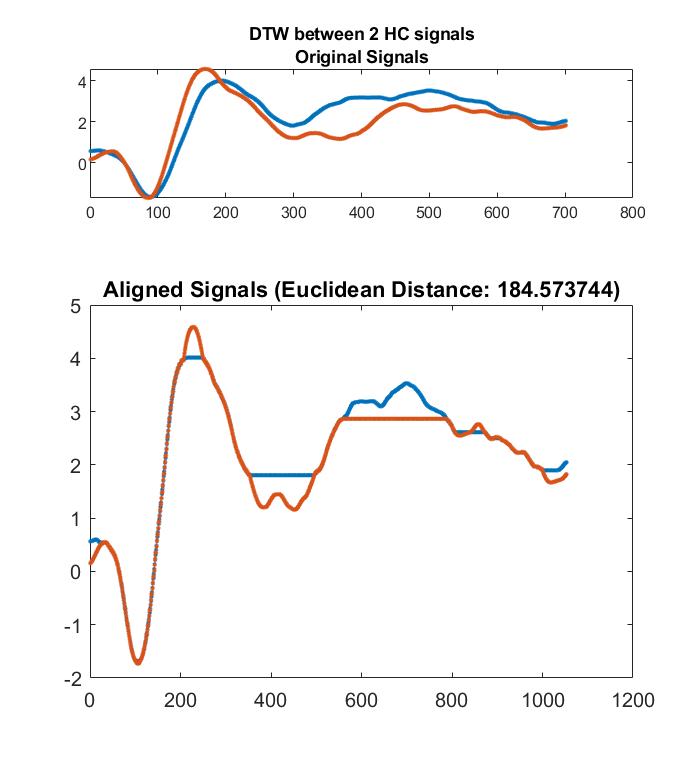

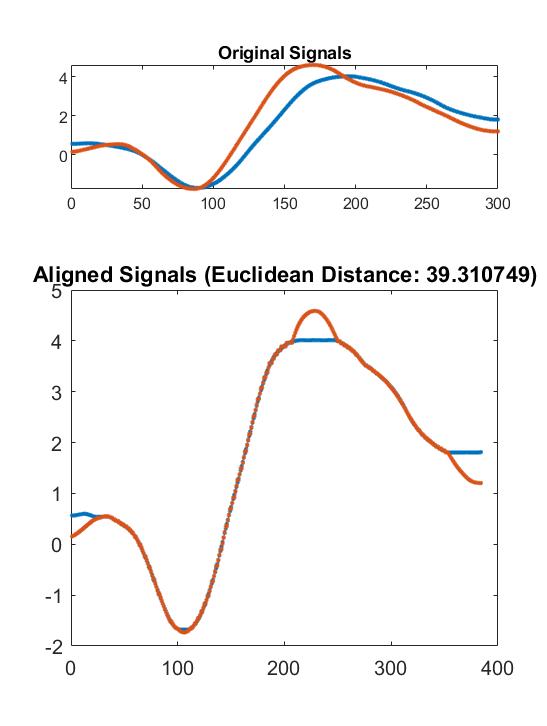


1. We can see for the same comparison among 2 Healthy Control signals the DTW for the whole epoch calculation and for the first 300ms of the ERP.

| **HC** | **SZ** |
| --- | --- |
| **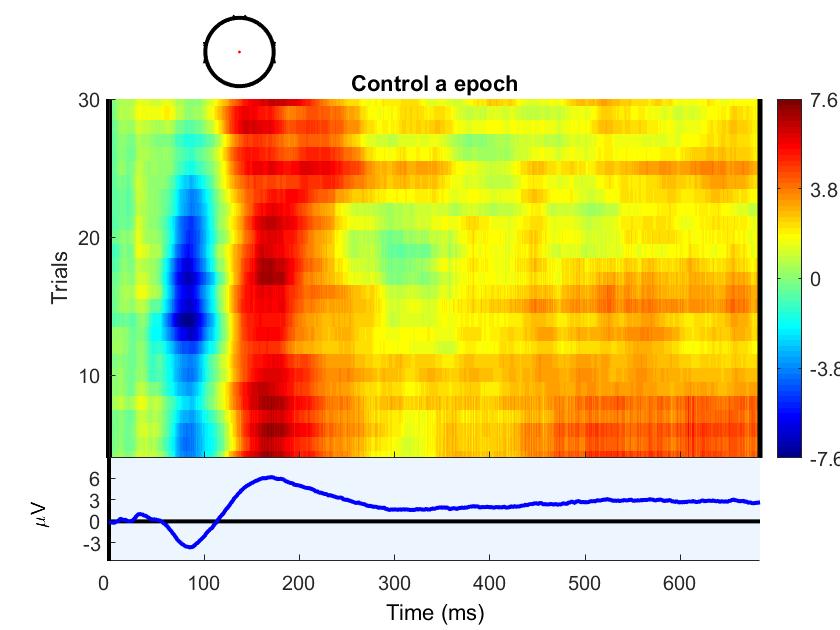** | **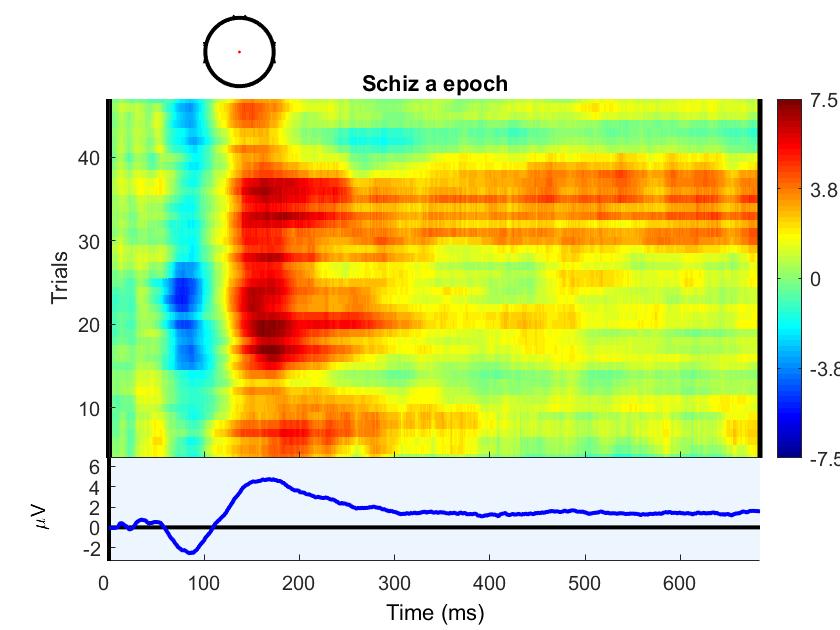** |
| **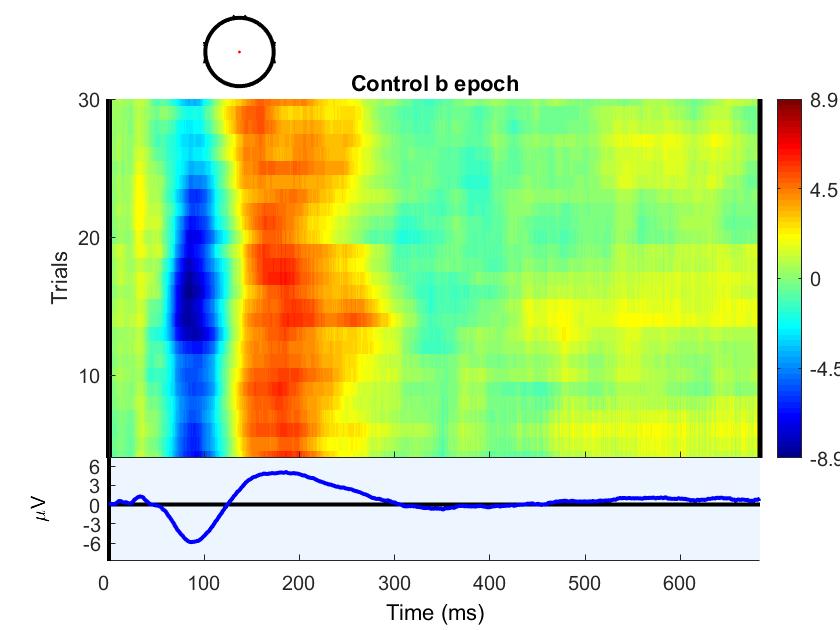** | **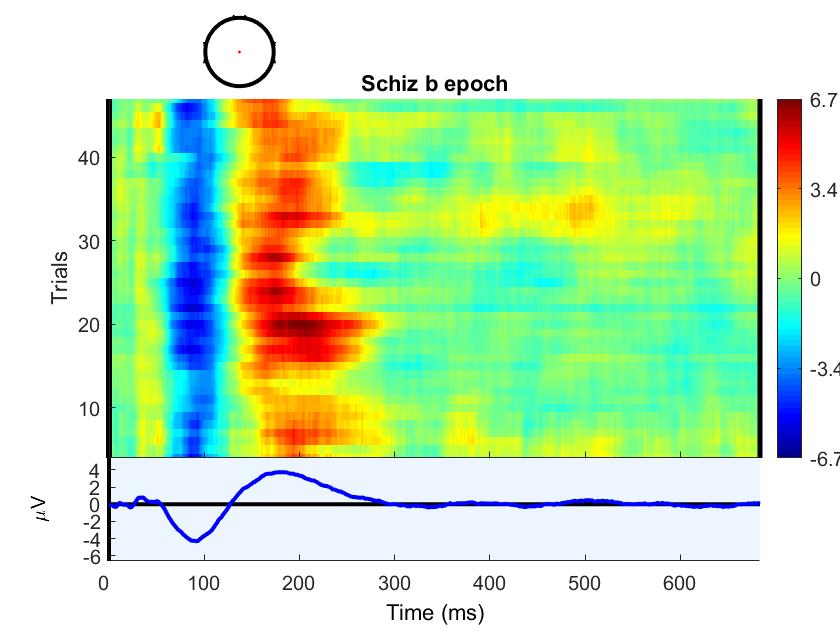** |

1. ERP activity for each trial indicates the potential differentiation among a and b epochs, for Healthy controls and Schizophrenic patients.

| HC | SZ |
| --- | --- |
| **Α**  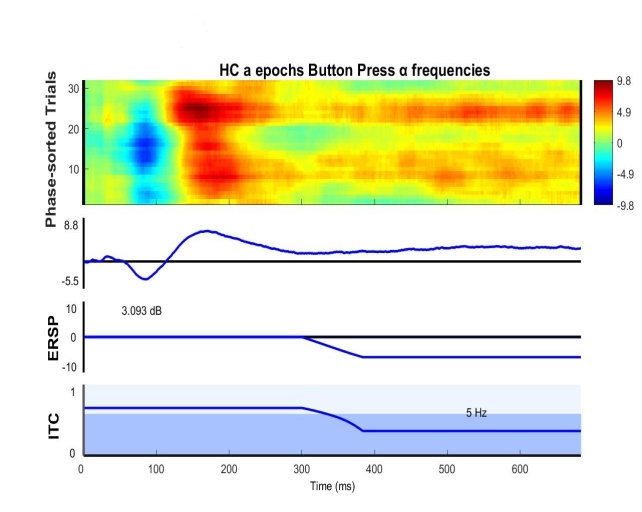 | *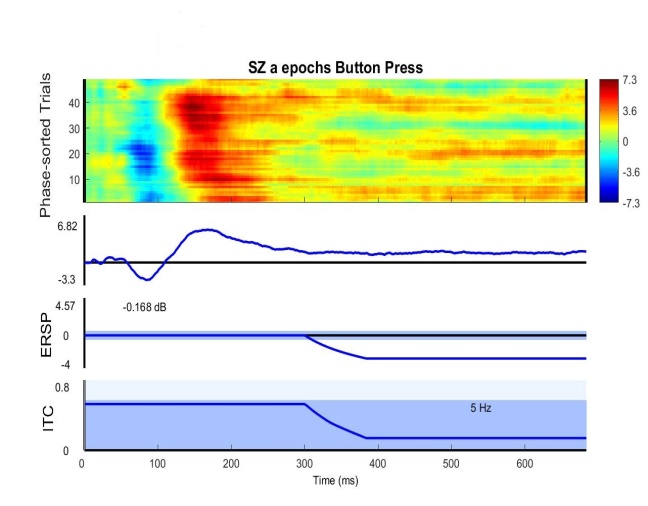* |
| **B**  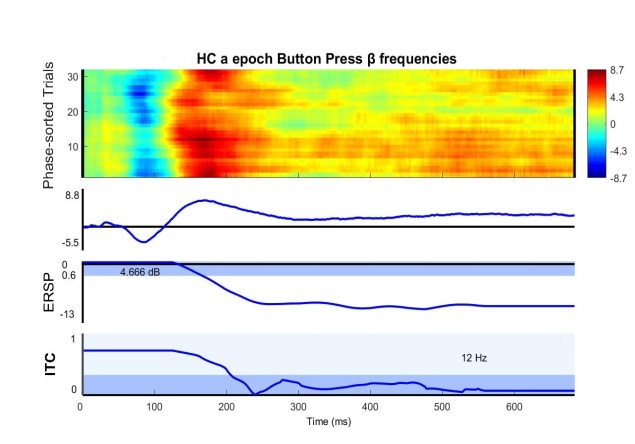 | 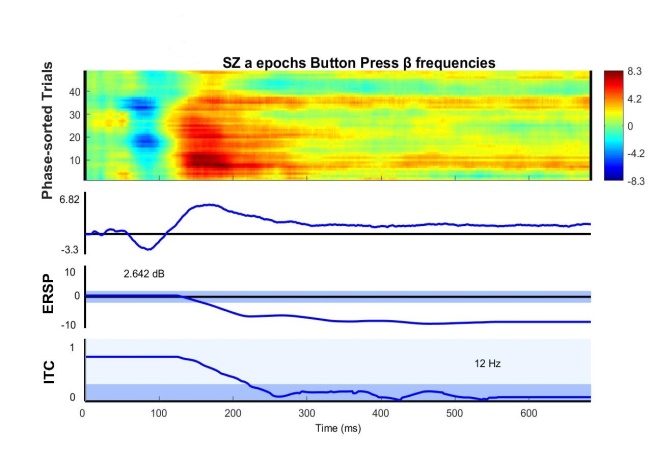 |
| **C**  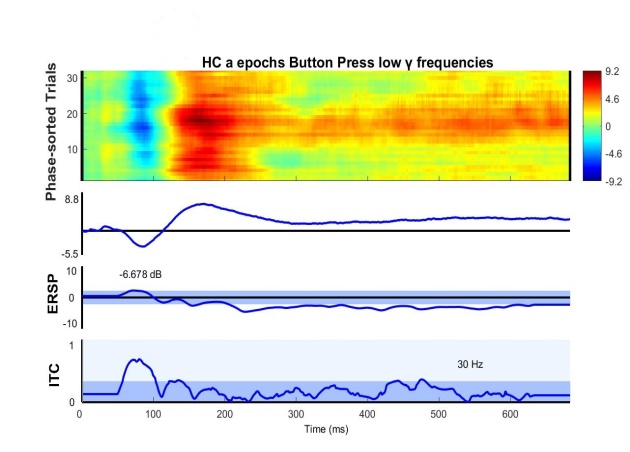 | 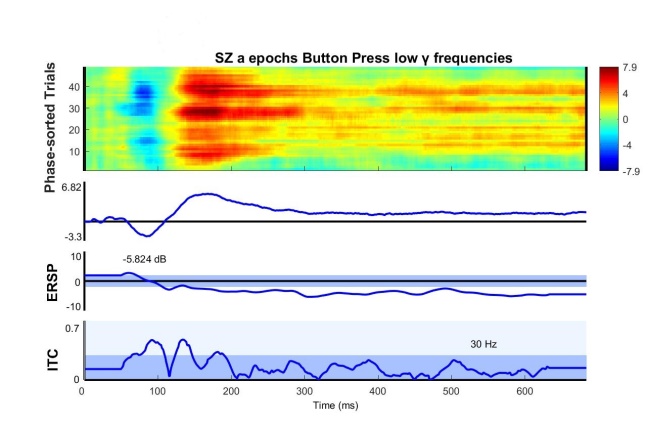 |
| **D**  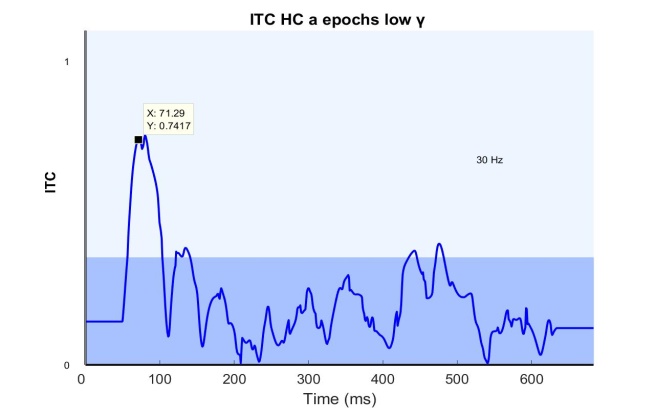 | 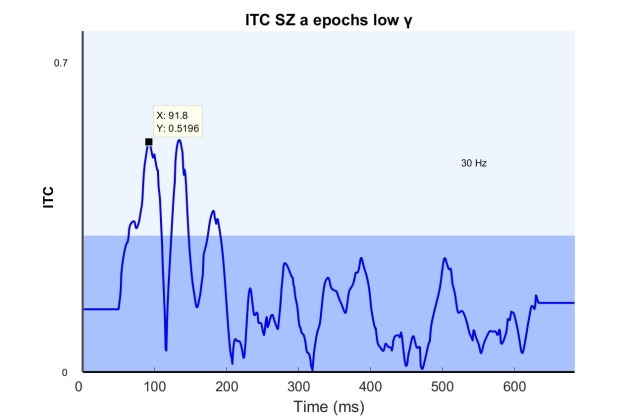 |
| 1. A. Inter-Trial Coherence (ITC) for HC and SZ for α frequencies (5-12Hz) B. ITC for HC and SZ for β frequencies (12-30Hz) C. ITC for HC and SZ for low γ frequencies (30-60Hz) D. ITC zoomed out at low γ for HC and SZ. | |
